# Supplementary material for: Judging the difficulty of perceptual decisions
Source: eLife. 2023 Nov 17;12:RP86892. doi: 10.7554/eLife.86892 (PMC10656101; doi:10.7554/eLife.86892)
Supplement: Supplementary file 1. [file elife-86892-supp1.docx]

| Subj | $\kappa$ | $u$ | $a$ | $d$ | $C_{0}$ | $\mu_{nd}$ |
| --- | --- | --- | --- | --- | --- | --- |
| 1 | 5.89 | 2.88 | 0.17 | -2.41 | -0.02 | 0.39 |
| 2 | 4.50 | 2.73 | 0.22 | -0.15 | 0.10 | 0.41 |
| 3 | 4.67 | 1.57 | 1.23 | 1.39 | -0.02 | 0.48 |
| 4 | 4.89 | 1.03 | 6.00 | 3.45 | 0.02 | 0.49 |
| 5 | 4.01 | 0.91 | 6.00 | 2.92 | -0.05 | 0.51 |
| 6 | 1.34 | 2.38 | 0.74 | 2.90 | 0.05 | 0.40 |
| 7 | 4.92 | 3.80 | 0.28 | -3.00 | -0.04 | 0.43 |
| 8 | 5.36 | 2.23 | 0.39 | 0.68 | 0.07 | 0.41 |
| 9 | 2.97 | 1.82 | -0.16 | 1.76 | 0.03 | 0.51 |
| 10 | 5.20 | 1.25 | 0.88 | 4.21 | 0.01 | 0.39 |
| 11 | 6.25 | 1.26 | 1.06 | 3.70 | 0.05 | 0.37 |
| 12 | 4.55 | 4.53 | 0.94 | -0.88 | -0.03 | 0.42 |
| 13 | 5.82 | 4.92 | 0.36 | -1.65 | 0.02 | 0.53 |
| 14 | 4.72 | 0.82 | 6.00 | 3.44 | 0.03 | 0.55 |
| 15 | 4.27 | 1.21 | 0.96 | 2.72 | -0.02 | 0.42 |
| 16 | 4.53 | 1.19 | 1.39 | 4.18 | -0.02 | 0.51 |
| 17 | 4.23 | 4.82 | 0.50 | -3.00 | 0.06 | 0.50 |
| 18 | 5.73 | 3.29 | 0.36 | -3.00 | -0.08 | 0.42 |
| 19 | 5.21 | 5.00 | 0.65 | -1.35 | 0.00 | 0.38 |
| 20 | 4.87 | 3.14 | -0.24 | 5.00 | 0.10 | 0.53 |
| ***Mean*** | 4.70 | 2.54 | 1.39 | 1.05 | 0.01 | 0.45 |
