# Supplementary material for: Judging the difficulty of perceptual decisions
Source: eLife. 2023 Nov 17;12:RP86892. doi: 10.7554/eLife.86892 (PMC10656101; doi:10.7554/eLife.86892)
Supplement: Supplementary file 2. [file elife-86892-supp2.docx]

| Subj | $\kappa$ | $u$ | $a$ | $d$ | $\mu_{nd}$ |  |
| --- | --- | --- | --- | --- | --- | --- |
| 1 | 3.98 | 1.22 | 5.00 | 1.31 | 0.25 |  |
| 2 | 3.65 | 1.50 | 4.64 | 1.54 | 0.19 |  |
| 3 | 2.97 | 0.97 | -1.02 | -0.49 | 0.40 |  |
| 4 | 3.88 | 1.54 | -0.81 | -0.13 | 0.36 |  |
| 5 | 3.82 | 1.18 | 2.45 | 0.73 | 0.34 |  |
| 6 | 1.94 | 1.46 | 3.34 | 1.90 | 0.38 |  |
| 7 | 3.20 | 1.63 | 1.08 | 1.11 | 0.32 |  |
| 8 | 3.53 | 1.18 | 2.47 | 4.00 | 0.19 |  |
| 9 | 2.15 | 0.72 | -1.50 | -1.75 | 0.42 |  |
| 10 | 2.64 | 1.29 | 4.78 | 1.45 | 0.27 |  |
| 11 | 2.40 | 1.36 | 4.98 | 1.94 | 0.11 |  |
| 12 | 2.76 | 1.63 | 1.56 | 1.67 | 0.32 |  |
| 13 | 2.50 | 1.39 | 4.85 | 1.55 | 0.21 |  |
| 14 | 2.92 | 1.36 | 1.15 | 1.93 | 0.24 |  |
| 15 | 3.76 | 1.12 | 4.79 | 1.87 | 0.19 |  |
| 16 | 2.81 | 1.83 | 4.98 | 1.93 | 0.10 |  |
| 17 | 3.30 | 0.76 | 4.16 | 1.87 | 0.43 |  |
| 18 | 3.06 | 1.25 | 4.78 | 1.80 | 0.23 |  |
| 19 | 3.23 | 1.19 | 4.19 | 0.78 | 0.34 |  |
| 20 | 3.04 | 1.22 | 4.50 | 1.58 | 0.30 |  |
| ***Mean*** | 3.08 | 1.29 | 3.02 | 1.33 | 0.28 |  |
