# Supplementary material for: Judging the difficulty of perceptual decisions
Source: eLife. 2023 Nov 17;12:RP86892. doi: 10.7554/eLife.86892 (PMC10656101; doi:10.7554/eLife.86892)
Supplement: Supplementary file 3. [file elife-86892-supp3.docx]

| Subj | $\kappa$ | $u$ | $a$ | $d$ | $\mu_{nd}$ |  |
| --- | --- | --- | --- | --- | --- | --- |
| 1 | 6.37 | 0.97 | 4.46 | 1.16 | 0.34 |  |
| 2 | 5.21 | 1.11 | 3.73 | 1.37 | 0.36 |  |
| 3 | 7.78 | 0.59 | 4.99 | 3.40 | 0.39 |  |
| 4 | 5.97 | 1.00 | -0.87 | -0.77 | 0.38 |  |
| 5 | 6.24 | 2.31 | 1.59 | -0.21 | 0.34 |  |
| 6 | 4.20 | 1.59 | 1.52 | 1.72 | 0.34 |  |
| 7 | 5.87 | 2.13 | 0.77 | -0.06 | 0.39 |  |
| 8 | 4.87 | 2.24 | 0.42 | -0.43 | 0.20 |  |
| 9 | 5.20 | 1.30 | -0.14 | 1.05 | 0.41 |  |
| 10 | 6.40 | 1.23 | 4.13 | 1.37 | 0.34 |  |
| 11 | 5.14 | 1.25 | 4.36 | 1.75 | 0.14 |  |
| 12 | 4.70 | 2.03 | 1.12 | 0.88 | 0.33 |  |
| 13 | 6.25 | 1.14 | 4.25 | 1.46 | 0.47 |  |
| 14 | 4.88 | 2.30 | 0.76 | 0.02 | 0.23 |  |
| 15 | 5.65 | 0.87 | 4.88 | 1.54 | 0.26 |  |
| 16 | 4.38 | 1.47 | 4.64 | 1.70 | 0.24 |  |
| 17 | 5.21 | 0.87 | 0.91 | 1.34 | 0.42 |  |
| 18 | 4.89 | 0.97 | 5.00 | 1.61 | 0.33 |  |
| 19 | 5.05 | 1.05 | 3.17 | 0.67 | 0.40 |  |
| 20 | 5.07 | 1.41 | 0.81 | 1.53 | 0.33 |  |
| ***Mean*** | 5.47 | 1.39 | 2.52 | 1.06 | 0.33 |  |
