# Supplementary material for: Judging the difficulty of perceptual decisions
Source: eLife. 2023 Nov 17;12:RP86892. doi: 10.7554/eLife.86892 (PMC10656101; doi:10.7554/eLife.86892)
Supplement: Supplementary file 4. [file elife-86892-supp4.docx]

| Subj | $\kappa$ | $u$ | $a$ | $d$ | $\mu_{nd}$ | $B_{mini}$ |
| --- | --- | --- | --- | --- | --- | --- |
| 1 | 7.10 | 0.87 | 2.52 | 1.43 | 0.32 | 1.20 |
| 2 | 4.89 | 0.98 | 4.72 | 1.29 | 0.28 | 1.34 |
| 3 | 7.16 | 0.63 | -0.85 | -1.44 | 0.37 | 0.73 |
| 4 | 6.00 | 0.99 | 4.06 | 1.47 | 0.37 | 0.18 |
| 5 | 5.99 | 1.84 | 2.33 | 0.16 | 0.34 | 0.66 |
| 6 | 3.73 | 1.76 | 1.36 | 1.40 | 0.30 | 1.40 |
| 7 | 6.11 | 1.74 | 1.18 | 0.42 | 0.39 | 0.95 |
| 8 | 4.73 | 1.15 | 0.58 | 1.80 | 0.19 | 1.12 |
| 9 | 3.97 | 0.69 | 4.90 | 3.72 | 0.42 | 0.32 |
| 10 | 6.16 | 1.26 | 3.45 | 1.34 | 0.32 | 1.17 |
| 11 | 5.11 | 1.17 | 4.54 | 1.68 | 0.12 | 1.41 |
| 12 | 4.99 | 2.05 | 1.26 | 0.84 | 0.35 | 1.25 |
| 13 | 5.79 | 1.17 | 2.66 | 1.47 | 0.43 | 1.22 |
| 14 | 5.21 | 1.56 | 1.03 | 0.95 | 0.25 | 0.97 |
| 15 | 5.70 | 0.86 | 4.56 | 1.77 | 0.21 | 1.03 |
| 16 | 4.49 | 1.49 | 3.69 | 1.64 | 0.13 | 1.82 |
| 17 | 3.50 | 2.72 | 2.70 | -0.79 | 0.41 | 0.78 |
| 18 | 4.92 | 1.34 | 5.00 | 1.43 | 0.36 | 0.16 |
| 19 | 5.11 | 3.16 | 1.92 | -0.17 | 0.33 | 0.75 |
| 20 | 5.34 | 0.90 | 4.83 | 1.61 | 0.35 | 1.16 |
| ***Mean*** | 5.30 | 1.42 | 2.82 | 1.10 | 0.31 | 0.98 |
