# Supplementary material for: Judging the difficulty of perceptual decisions
Source: eLife. 2023 Nov 17;12:RP86892. doi: 10.7554/eLife.86892 (PMC10656101; doi:10.7554/eLife.86892)
Supplement: Supplementary file 5. [file elife-86892-supp5.docx]

| Subj | $\kappa$ | $u$ | $a$ | $d$ | $\mu_{nd}$ |  |
| --- | --- | --- | --- | --- | --- | --- |
| 1 | 15.41 | 0.65 | 5.00 | 1.07 | 0.46 |  |
| 2 | 14.00 | 0.84 | 4.57 | 1.22 | 0.41 |  |
| 3 | 15.18 | 0.52 | 0.85 | 2.30 | 0.42 |  |
| 4 | 14.49 | 0.56 | 1.57 | 3.09 | 0.41 |  |
| 5 | 15.16 | 0.86 | 3.17 | 0.43 | 0.39 |  |
| 6 | 11.71 | 2.74 | 0.92 | 0.18 | 0.28 |  |
| 7 | 14.14 | 1.67 | 0.98 | -0.07 | 0.44 |  |
| 8 | 12.98 | 1.13 | 0.96 | 0.99 | 0.24 |  |
| 9 | 14.27 | 0.67 | 0.66 | 1.62 | 0.44 |  |
| 10 | 14.71 | 0.96 | 2.70 | 1.20 | 0.38 |  |
| 11 | 13.70 | 0.92 | 5.00 | 1.59 | 0.24 |  |
| 12 | 12.98 | 1.44 | 1.32 | 0.78 | 0.45 |  |
| 13 | 14.84 | 0.84 | 4.87 | 1.29 | 0.53 |  |
| 14 | 13.35 | 2.03 | 0.87 | -0.32 | 0.30 |  |
| 15 | 15.48 | 0.70 | 2.64 | 1.73 | 0.30 |  |
| 16 | 12.61 | 1.10 | 4.23 | 1.56 | 0.36 |  |
| 17 | 14.51 | 1.41 | 0.74 | -0.57 | 0.45 |  |
| 18 | 13.14 | 0.74 | 2.99 | 1.54 | 0.38 |  |
| 19 | 13.17 | 1.60 | 2.44 | 0.13 | 0.38 |  |
| 20 | 14.15 | 0.85 | 2.45 | 1.32 | 0.43 |  |
| ***Mean*** | 14.00 | 1.11 | 2.45 | 1.05 | 0.38 |  |
