# Supplementary material for: Judging the difficulty of perceptual decisions
Source: eLife. 2023 Nov 17;12:RP86892. doi: 10.7554/eLife.86892 (PMC10656101; doi:10.7554/eLife.86892)
Supplement: Supplementary file 6. [file elife-86892-supp6.docx]

| Subj | $\kappa$ | $u$ | $a$ | $d$ | $\mu_{nd}$ |  |
| --- | --- | --- | --- | --- | --- | --- |
| 1 | 7.05 | 1.09 | 1.34 | 0.14 | 0.37 |  |
| 2 | 6.07 | 2.06 | 0.50 | -0.09 | 0.29 |  |
| 3 | 7.17 | 2.89 | 2.45 | 0.18 | 0.44 |  |
| ***Mean*** | 6.77 | 2.01 | 1.43 | 0.07 | 0.37 |  |
