# Supplementary material for: Judging the difficulty of perceptual decisions
Source: eLife. 2023 Nov 17;12:RP86892. doi: 10.7554/eLife.86892 (PMC10656101; doi:10.7554/eLife.86892)
Supplement: Supplementary file 7. — The high parameter for subject 2 implies that the decision process was not bounded. [file elife-86892-supp7.docx]

| Subj | $\kappa$ | $u$ | $a$ | $d$ |
| --- | --- | --- | --- | --- |
| 1 | 10.41 | 0.17 | 0.15 | 9.27 |
| 2 | 9.27 | 14.97 | 0.49 | 3.32 |
| 3 | 8.63 | 0.36 | 0.94 | 4.25 |
| ***Mean*** | 9.44 | 5.17 | 0.53 | 5.61 |
