# Supplementary material for: Judging the difficulty of perceptual decisions
Source: eLife. 2023 Nov 17;12:RP86892. doi: 10.7554/eLife.86892 (PMC10656101; doi:10.7554/eLife.86892)
Supplement: Supplementary file 8. [file elife-86892-supp8.docx]

| Subj | $\kappa$ | $u$ | $a$ | $d$ | $\mu_{nd}$ |
| --- | --- | --- | --- | --- | --- |
| 1 | 5.17 | 2.17 | 4.79 | 0.35 | 0.38 |
| 2 | 3.67 | 2.84 | 1.01 | 0.56 | 0.35 |
| 3 | 4.83 | 5 | 3.33 | 0.27 | 0.53 |
| ***Mean*** | 4.56 | 3.34 | 3.04 | 0.39 | 0.42 |
